# The cox-filter method identifies respective subtype-specific lncRNA prognostic signatures for two human cancers

**DOI:** 10.1186/s12920-020-0691-4

**Published:** 2020-02-05

**Authors:** Suyan Tian, Chi Wang, Jing Zhang, Dan Yu

**Affiliations:** 1 Division of Clinical Research, The First Hospital of Jilin University, 1Xinmin Street, Changchun, Jilin, 130021 People’s Republic of China; 2 Department of Biostatistics, College of Public Health, University of Kentucky, 800 Rose St, Lexington, KY 40536 USA; 3 Markey Cancer Center, University of Kentucky, 800 Rose St, Lexington, KY 40536 USA; 4 School of Life Science, 2699 Qianjin Street, Changchun, Jilin, 130012 People’s Republic of China; 5 Department of Otolaryngology Head and Neck Surgery, The Second Hospital of Jilin University, 218 Ziqiang Road, Changchun, Jilin, 130041 People’s Republic of China

**Keywords:** Long non-coding RNA (lncRNA), Prognostic signature, Head and neck squamous cell carcinoma (HNSCC), Esophageal cancer, Cox regression model

## Abstract

**Background:**

The most common histological subtypes of esophageal cancer are squamous cell carcinoma (ESCC) and adenocarcinoma (EAC). It has been demonstrated that non-marginal differences in gene expression and somatic alternation exist between these two subtypes; consequently, biomarkers that have prognostic values for them are expected to be distinct. In contrast, laryngeal squamous cell cancer (LSCC) has a better prognosis than hypopharyngeal squamous cell carcinoma (HSCC). Likewise, subtype-specific prognostic signatures may exist for LSCC and HSCC. Long non-coding RNAs (lncRNAs) hold promise for identifying prognostic signatures for a variety of cancers including esophageal cancer and head and neck squamous cell carcinoma (HNSCC).

**Methods:**

In this study, we applied a novel feature selection method capable of identifying specific prognostic signatures uniquely for each subtype – the Cox-filter method – to The Cancer Genome Atlas esophageal cancer and HSNCC RNA-Seq data, with the objectives of constructing subtype-specific prognostic lncRNA expression signatures for esophageal cancer and HNSCC.

**Results:**

By incorporating biological relevancy information, the lncRNA lists identified by the Cox-filter method were further refined. The resulting signatures include genes that are highly related to cancer, such as H19 and NEAT1, which possess perfect prognostic values for esophageal cancer and HNSCC, respectively.

**Conclusions:**

The Cox-filter method is indeed a handy tool to identify subtype-specific prognostic lncRNA signatures. We anticipate the method will gain wider applications.

## Background

Esophageal cancer is a cancer of the esophagus, the hollow tube that carries foods and liquids from throat to stomach. The causes of esophageal cancer are unclear, but it is commonly believed that both environmental and genetic factors play roles in its initiation and progression [1]. For instance, smoking, heavy alcohol consumption, obesity, and damage to the esophagus from acid reflux (Barrett esophagus) are thought to increase the risk of developing esophageal cancer, while, the tendency of familial aggregation for esophageal cancer suggests that genetic components are of crucial importance. The most common histological subtypes of esophageal cancer are squamous cell carcinoma (ESCC) and adenocarcinoma (EAC). As far as prognosis is concerned, no evidence suggests any substantial difference between these two subtypes. Nevertheless, a study by The Cancer Genome Atlas research group [2] has demonstrated that non-marginal differences with regard to gene expression and somatic alteration exist between ESCC and EAC. Consequently, biomarkers that hold prognostic value for these two subtypes are expected to be distinct, at least to some extent.

Head and neck squamous cell carcinoma (HNSCC) develops in mucous membranes of the mouth, nose and throat. Hypopharyngeal squamous cell carcinoma (HSCC), which originates in mucosa of the hypopharynx and accounts for approximately 3% of HNSCC cases, has one of the poorest prognoses among HNSCC patients [3]. Laryngeal squamous cell cancer (LSCC) accounts for relatively more HNSCC cases and has a better prognosis compared to HSCC even though the initial sites of these two diseases are anatomically very close. LSCC originates in the larynx, whereas HSCC originates in the lower part of the throat near the larynx (i.e., the hypopharynx). Therefore, finding molecular markers that can distinguish between the two subtypes is crucial for survival prediction.

Long non-coding RNAs (lncRNAs) are a class of RNA molecules that have a length of more than 200 nucleotides and are without protein-coding capacity [4]. Therefore, lncRNAs have previously been regarded as transcriptional “junk.” Nowadays, paramount investigations have demonstrated that lncRNAs can serve as novel biomarkers and therapeutic targets in complex diseases such as cancer. Identification of lncRNA signatures is in demand and usually requires the help of a feature selection method. The primary aims of feature selection are to reduce the number of features (e.g., genes or metabolites) under consideration to a manageable size, thus speeding up the learning process and facilitating biological interpretation and experimental validation [5].

Applying feature selection to lncRNA (vs mRNA) data might achieve better model parsimony because mRNA-based studies obtain signatures with a limited number of genes, and because the expression levels of lncRNAs are usually lower than those of mRNAs (thus less differentially expressed lncRNAs can be identified). Studies that aim to identify lncRNA signatures for esophageal cancers and HNSCC have increased dramatically. For example, studies by Cao et al. [6], Wang et al. [7] and Yao et al. [8] specifically aimed to identify lncRNA expression signatures with prognostic value for HNSCC patients, while several studies [9–12] identified relevant lncRNA signatures for esophageal cancer. Nevertheless, those studies usually considered HNSCC or esophageal cancer as a whole or only focused on one specific subtype.

In this study, we applied a novel feature selection method – the Cox-filter method [13] – to the cancer genome atlas (TCGA) esophageal cancer and HNSCC RNA-Seq data, with the objectives of constructing subtype-specific prognostic lncRNA expression signatures for EC and HNSCC. Precision medicine for those patients will only be possible once subtype-specific prognostic signatures become available.

## Materials and methods

### Experimental data

The lncRNA expression values, namely, FPKM (fragments per kilo-bases per million) for HNSCC were retrieved from the TANRIC (The Atlas of ncRNA in Cancer) database [14], version 1.0.6 (https://www.tanric.org/), which was last updated on 07/29/2015. Then the corresponding clinical information was retrieved from the the Genomic Data Commons (https://gdc.cancer.gov) by matching the barcode IDs of samples in the TANRIC database [14] with those in the TCGA database. Patients without information on overall survival (OS), age, gender, pathological tumor stage and histological subtype were discarded. Only patients with LSCC and HSCC were retained for analysis. If the sum of FPKM values of lncRNA expression across all samples (LSCC and HSCC patients combined) was < 4, they were deleted. Finally, log 2 transformations on (FPKM counts + 1) were carried out, providing a better approximation to a normal distribution.

For the esophageal cancer study, both the expression profiles (RNA-Seq data) of TCGA ESCA cohort and clinical information such as overall survival time were downloaded from the Genomic Data Commons. Subsequently, the lncRNAs were collected by mapping the Ensemble IDs of RNA-Seq data to those in the TANRIC database [14] (given that the ESCA cohort is not included in the TANRIC database) so that expression profiles of lncRNAs were obtained.

The ratio of LSCC and HSCC is extremely high (89:6) while that for ESCC to EAC is very close to 1 (81:83), which represents the two extreme cases (huge imbalance of sample ratios versus perfect balance of sample ratios). Hence, using these two datasets, it is possible to examine the influence of subgroup size imbalance on the performance of a feature selection algorithm. The demographical characteristics of these two datasets are presented in Table 1.

**Table 1:** Characteristics of head and neck squamous cell carcinoma and esophageal cancer data

	Patients (#)	Deaths (#)	Median survival time (days)	p-value (log-rank test)
Esophageal cancer				
Squamous Cell Carcinoma [ESCC]	81	29	763	
Adenocarcinoma [EAC]	83	38	801	0.721
Head and Neck Squamous Cell Carcinoma [HNSCC]				
Laryngeal [LSCC]	89	25	1838	
Hypopharyngeal [HSCC]	6	2	–	0.839

The log-rank tests indicated histological subtype but had no prognostic value for esophageal cancer or HNSCC. For esophageal cancer, this is consistent with previous results. For HNSCC the discrepancy may be attributable to the small sample size of HSCC subtype

## Statistical methods

The Cox-filter method proposed by Tian et al. [13] screens genes one by one according to the significance level of the corresponding coefficients in a Cox model. Under the two-class cases (the model can easily be extended to multiple-class cases), the corresponding Cox model may be written as,

$$ {\lambda}_{\mathrm{i}\mathrm{jg}}\left(\mathrm{t}\right)={\lambda}_{0\mathrm{g}}\left(\mathrm{t}\right)\exp \Big({\upbeta}_{1\mathrm{g}}{\mathrm{I}}_{\mathrm{i}}\left(\mathrm{j}={\mathrm{c}}_2\right)+{\upbeta}_{2\mathrm{g}}{\mathrm{X}}_{\mathrm{i}\mathrm{jg}}+\left({\upbeta}_{1\mathrm{g}}{\mathrm{I}}_{\mathrm{i}}\left(\mathrm{j}={\mathrm{c}}_2\right)\times {\mathrm{X}}_{\mathrm{i}\mathrm{jg}}\right) $$

Tian et al. [13] provided a detailed description of the definitions of parameters (i.e., βs and λs) and a graphical illustration of all possible scenarios; those details are not presented here. For the current study, the features under consideration are lncRNAs, subtype-specific prognostic lncRNAs were those for which either β_2g_ or (β_2g_ + β_3g_) is significantly different from zero. More specifically, β_2g_ ≠ 0 implies that lncRNA *g* has a prognostic value for subgroup c_1_ while (β_2g_ + β_3g_) ≠ 0 implies lncRNA *g* has a prognostic value for subgroup c_2_. Therefore, β_2g_ and β_3g_ are the parameters of interest and their significance levels determine if subtype-specific lncRNAs exist.

### Statistical language and packages

All statistical analyses were carried out in the R language, version 3.5 (www.r-project.org).

## Results

**Fig. 1:**
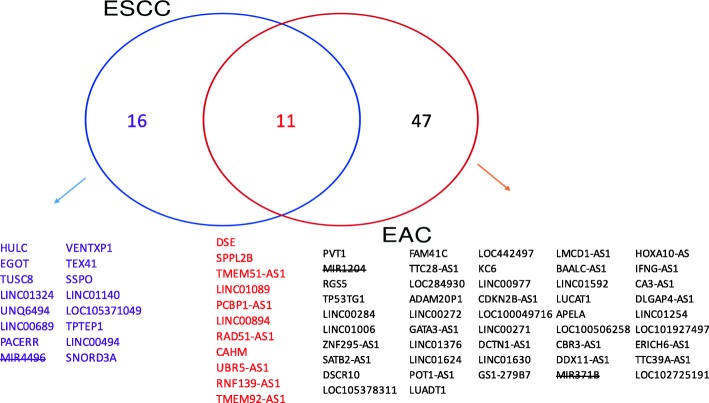
Venn-diagram illustrating EAC- and ESCC-specific prognostic lncRNAs. Gene symbols of microRNAs that were misclassified as lncRNAs are crossed out. EAC: esophageal adenocarcinoma; ESCC: esophageal squamous cell carcinoma

By applying the Cox-filter model to esophageal cancer data and setting the cutoff of adjusted *p*-values for these linear coefficients at 0.05, we identified 200 lncRNAs that have prognostic values for EAC and 96 for ESCC. Among them, there were 46 overlaps. We searched the GeneCards database for their biological relevance. For EAC, after removing 19 genes that are not be recognized by the GeneCards database (www.genecards.org), 58 lncRNAs were indicated to be directly related to cancers. For ESCC, 19 lncRNAs are unrecognizable as well. Among the remaining 77 lncRNAs, 27 of them were directly related to cancers. A Venn-diagram (Fig. 1) was made and the gene symbols were given, stratified by EAC-specific lncRNAs, ESCC-specific lncRNAs and overlapped lncRNAs between two subtypes. Among these unique 74 lncRNAs, 44 were regarded as being differentially expressed between cancer tissues and normal tissues.

**Fig. 2:**
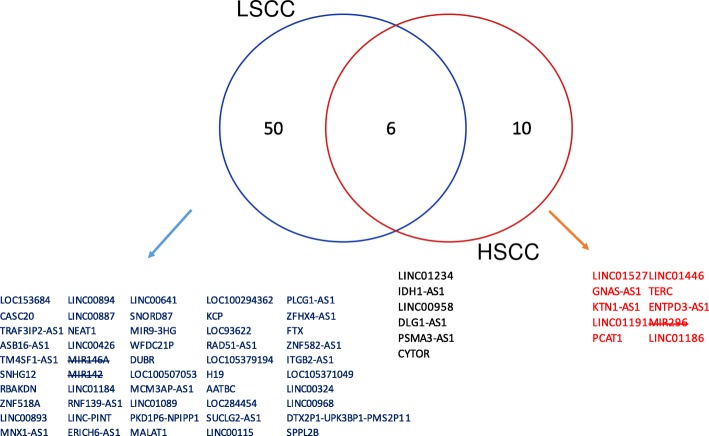
Venn-diagram illustrating LSCC-specific prognostic lncRNAs and HSCC-specific prognostic lncRNAs. Gene symbols of microRNAs that were misclassified as lncRNAs are crossed out. LSCC: laryngeal squamous cell cancer; HSCC: hypopharyngeal squamous cell cancer

For HNSCC, using a cutoff of 0.05 for adjusted *p*-values the Cox-filter method identified 126 LSCC lncRNAs (20 non-identifiable in the GeneCards database) and 89 HSCC lncRNAs (30 of which are non-identifiable in the GeneCards database). Fifty-six were directly related to cancers for LSCC and 16 for HSCC. Among these lncRNAs, 6 lncRNAs were shared by these two subtypes, and 44 lncRNAs were regarded as being differentially expressed between cancer tissues and normal tissues. Figure 2 presents gene symbols of those lncRNAs. From the gene symbols given in Figs. 1 and 2, we observed several microRNAs (e.g., MIR146A and MIR 296) that were mistakenly recognized as lncRNAs by the TANRIC database. Since TANRIC has not been updated since its initiation, it is natural to expect such errors. In the following results, those microRNAs were removed manually.

## Discussion

In this study, Pvt1 oncogene (PVT1) with a confidence score of 25.4 is ranked on the second place for the EAC-specific prognostic lncRNAs. Based on the strategy of competitive endogenous RNA (ceRNA) networks [15], overexpression of PVT1 correlates with a poor prognosis [16] or a fast tumor progression [17] in esophageal cancer patients or in ESCC [18] In this study, PVT1 was indicated as an EAC-specific lncRNA since it does not belong to the intersection set between lncRNAs for these two subtypes.

CDKN2B antisense RNA 1 (CDKN2B-AS1), also known as ANRIL, was on the top of this list (i.e., cancer related EAC-specific prognostic lncRNAs), however, only three studies [19–21] have addressed its association with esophageal cancer. While the first two studies explored the association between CDKN2B-AS1 and esophageal cancer by way of genetic mutations, the third did so from the prospective of expression level. Other than esophageal cancer, CDKN2B-AS1 had been linked to a variety of cancer types such as acute lymphoblastic leukemia [22], gastric cancer [20, 23] and hepatocellular carcinoma (HCC) [24]. For other top-ranked lncRNAs, Yoon et al. [25] have demonstrated that LUCAT1 was over-expressed in tumor issues compared to paired normal tissues and may promote carcinogenesis of ESCC. Another recent study [26] has shown that up-regulation of CBR3-AS1 promoted cell proliferation and was positively correlated with pathologic stages of ESCC. Lastly, despite the absence of literature suggesting that TP53TG plays any role in the development and progression of esophageal cancer, this lncRNA can suppress tumor growth and is of importance for the correct response of P53 to DNA damage [27]. In addition, the association of TP53TG with other cancer types such as glioma and lung caner has been reported in previous studies.

Besides the lower prevalence of lncRNA studies on EAC, another possible explanation for the links of top-5-ranked lncRNAs with ESCC instead of EAC is that racial disparities of ESCC between Asian and Caucasian populations existed at the molecular level [28]. Then, it is natural to observe a link between PVT1 and ESCC during the literature mining considering those studies were all carried out in East Asia. In contrast, our work is based on the TCGA RNA-Seq data in which most patients are Whites.

**Fig. 3:**
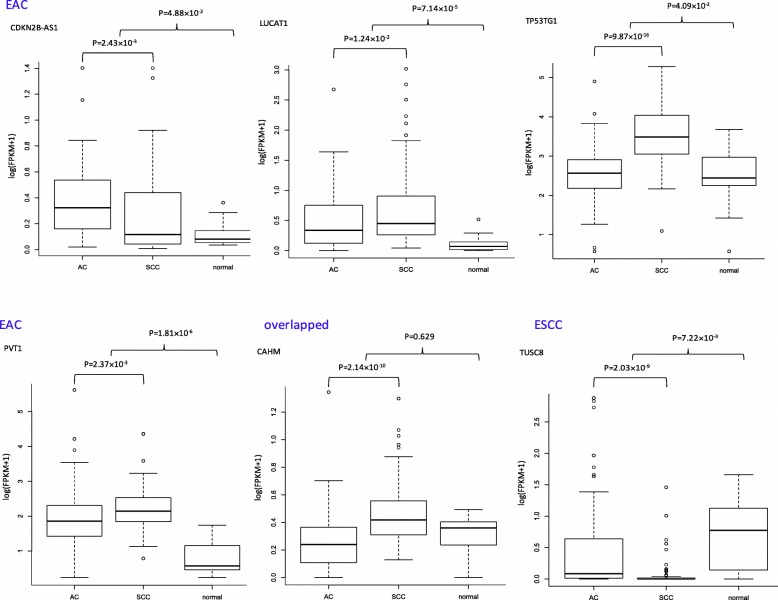
Box-plots illustrating the expression levels of 6 differentially expressed lncRNAs between EAC and ESCC (which have a Wilcoxon test *p*-value < 0.05). Among them, 5 lncRNAs may be regarded as differentially expressed lncRNAs between esophageal cancer and normal controls (which have a corresponding Wilcoxon test *p*-value < 0.05 as well). EAC: esophageal adenocarcinoma; ESCC: esophageal squamous cell carcinoma

**Fig. 4:**
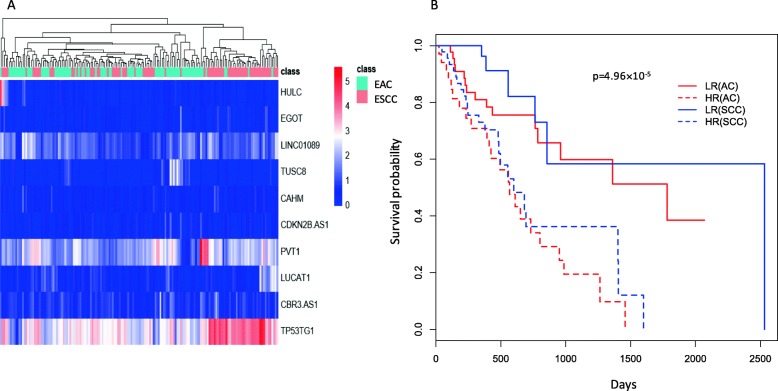
Discriminative value and prognostic value of the top 10 directly-related-to-cancer lncRNAs identified by the Cox-filter method for the esophageal cancer application. **a** Heat-map. **b** Kaplan-Meier curves. Based on the risk scores calculated using a multivariate Cox regression model, the samples were divided into a high- and low-risk of death groups. From these two plots, it was observed that while the lncRNAs possessed little information for segmentation of EAC and ESCC, they can distinguish the high- and low-risk groups perfectly well. In the Kaplan-Meier plot the log-rank p-value was also given. EAC: esophageal adenocarcinoma; ESCC: esophageal squamous cell carcinoma; LR: low-risk group; HR: high-risk group

On the other hand, for the top 5 directly-related-to-cancer lncRNAs for the ESCC, only two studies provided experimental supports on the association of HULC [29] and EGOT [30] with esophageal cancer. For the remaining three lncRNAs – LINC01089, TUSC8 and CAHM — the LncRNADisease2 database [31] used computational methods and predicted they are associated with gastric cancer. Even though the identified lncRNAs are related to a variety of cancers, more focus on their correlations with ESCC and EAC are in demand. The expression levels of those 10 lncRNAs were compared between ESCC and EAC, between esophageal cancer tissues and normal tissues using Wilcoxon tests. Among them, 6 (4 were specific for EAC, 1 for ESCC and 1 shared by both subtypes) had a corresponding *p*-value < 0.05 and may be considered as the differentially expressed lncRNAs between EAC and ESCC (Fig. 3). All these 6 lncRNAs except CAHM had corresponding Wilcoxon test *p*-values < 0.05 in the comparison of tumor tissues and normal tissues as well (Fig. 3). Nevertheless, as shown in Fig. 4a, these 10 lncRNAs hold very limited discriminative capacity to separate EAC from ESCC. In contrast, they can predict the prognosis status perfectly. In Fig. 4b, Kaplan-Meier curves were plotted for high-risk and low-risk groups (stratified according to the estimated risk scores of the multivariate Cox-regression model with these 10 lncRNAs as covariates), and then a log-rank test was performed to compare these survival curves. From Fig. 4b, we observed that within each subtype, the difference between the high-risk and low-risk groups was significant while within each risk group (between subtype), the difference was less or not significant. This result is expectable given that the outcomes (i.e., dependent variables) considered in the segmentation of subtypes and prognosis prediction are distinct.

Among the overlapped 11 lncRNAs, in addition to that CAHM was experimentally validated to be associated with colorectal cancer by a qPCR study [32] and astrocytoma [33] by a microarray study, TMEM51-AS1 was with chromophobe renal cell carcinoma [34] and liver cancer [35] by qPCR studies, RAD51-AS1 was with only ovarian epithelial cancer [36], RNF139-AS1 was with only astrocytoma [37] and LINC01089 with breast cancer [38] by qPCR and astrocytoma [33] by a microarray study, all except DSE and SPPL2B (which is not recorded on LncRNADisease2 database) were predicted to be correlated with a variety of cancers such as gastric cancer by the LncRNADisease2 database. Further studies are warranted to investigate the roles that the identified lncRNAs (including overlapped ones and unique-to-subtype ones by integrating the Cox-filter method and biological relevancy) may play during the development and progression of esophageal cancer.

For LSCC prognostic lncRNAs, H19, MALAT1, NEAT1, CYTOR and SNHG12 were ranked as the first five of this directly-related-to-cancer list. For HSCC, TERC, PCAT1, CYTOR, LINC01234 and LINC00958 made to the list. H19 is a well-known oncogene and acts as a driving force in a variety of cancers. For HNSCC specifically, a study by Guan et al. [39] demonstrated that overexpression of H19 is associated with tumor recurrence and poor prognosis by performing an experiment including 62 HNSCC patients (46 with LSCC and 14 with HSCC). A very recent study [40] also showed that the expression level of H19 was higher in patients with metastasized (vs non-metastasized) tongue squamous cell carcinoma, and was higher in tumor cells than normal squamous cells.

MALAT1 was found to be overexpressed in tumor tissues of oral squamous cell carcinoma (OSCC) patients by a real-time PCR experiment carried out by Zhou et al. [41]. Chang et al. [42] showed that inhibition of MALAT1 can prevent OSCC proliferation whereas its overexpression can promote OSCC. According to the ceRNA network, MALAT1 is a microRNA sponge of miR-125b of which STAT3 is predicted as a binding target. In addition, two studies [43, 44] provided experimental supports for the association of MALAT1 and tongue squamous cell carcinoma. Using qRT-PCR, Wang et al. [45] examined and compared the expression level of NEAT1 in LSCC and adjacent non-neoplastic tissues and showed that NEAT1 was significantly over-expressed in LSCC. Hence, they concluded that “NEAT1 plays an oncogenic role in the tumorigenesis of LSCC.”

CYTOR, also known as LINC00152, was proved experimentally to be associated with progression and prognosis of tongue squamous cell carcinoma [46] and HNSCC [47]. Using TCGA RNA-Seq data and some bioinformatics tools, Guo et al. [48] identified CYTOR as an HNSCC-associated lncRNA and determined that its expression is positively correlated with lymph node metastasis and risk of death. Subsequently, its function was explored by cell-based experiments which suggested that CYTOR inhibited cell apoptosis after the treatment with chemotherapeutic drug diamminedichloroplatinum (DDP). Furthermore, acting as the microRNA sponge of miR-19-5p that combines with the 3’UTR region of WWP1, overexpression of SNHG12 may promote proliferation and invasion of LSCC [49]. In our analysis, CYTOR was shared by both LSCC and HSCC subtypes.

Even though no experimental evidence or computational prediction links TERC with HNSCC in the LncRNADisease2.0 database [31], literature mining in the PubMed database identified several studies to support their association. For LSCC specifically, Liu et al. [50] detected TERC gene amplification in precancerous and cancerous tissues using fluorescent in situ hybridization. In a recent study [51], the expression values of PCAT1 in paired HNSCC tissues and adjacent non-tumor tissues were measured using qRT-PCR. The results showed that PCAT1 was over-expressed in the tumor tissues, which consisted with the results given by the online bioinformatics tool, GEPIA (http://gepia.cancer-pku.cn). In addition, that study also proved that after the knockdown of PCAT1, p38 MAPK and apoptosis signal-regulating kinase 1 which induced Caspase 9 and PART mediated apoptosis were activated.

For the last two HSCC-specific lncRNAs, namely, LINC01234 and LINC00958, no evidence has been found to link them with HNSCC in either the LncRNADisease2 database (experimentally or computationally) or the PubMed literature search. Both of these genes overlapped the HSCC and LSCC subtypes. Likewise, for the final 3 overlapped lncRNAs, no support for a link with HNSCC can be found. Further studies are warranted.

**Fig. 5:**
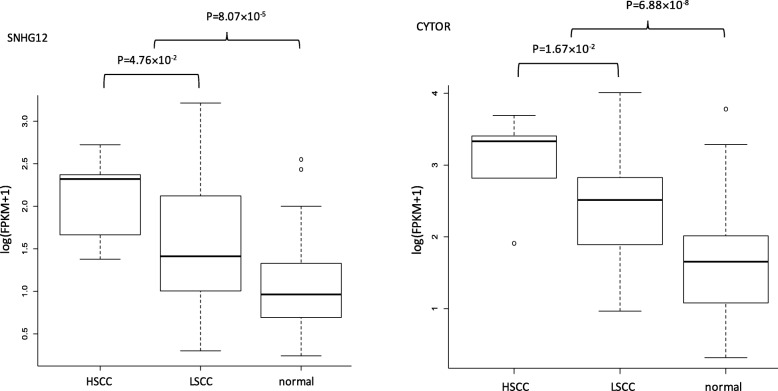
Box-plots illustrating the expression levels of 2 differentially expressed lncRNAs between LSCC and HSCC (which have a Wilcoxon test p-value < 0.05). Because the sample size of HSCC is very small, only two lncRNAs barely made the significance level of 0.05, which were differentially expressed lncRNAs between cancer tissues and normal tissues as well. LSCC: laryngeal squamous cell cancer; HSCC: hypopharyngeal squamous cell cancer

**Fig. 6:**
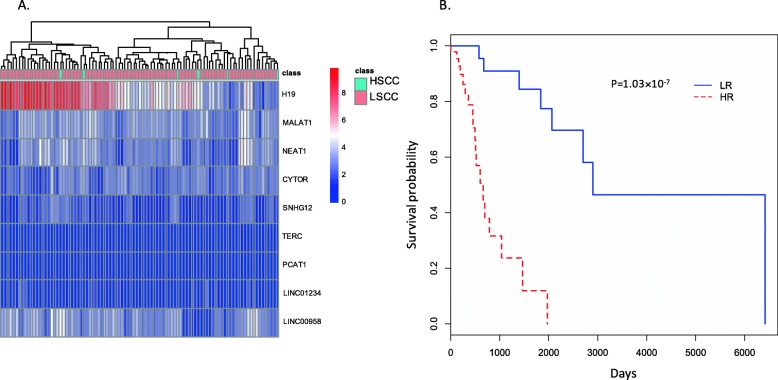
Discriminative value and prognostic value of 9 top directly-related-to-cancer lncRNAs identified by the Cox-filter method for the head and neck cancer. **a** Heat-map of these lncRNAs. **b** Kaplan-Meier curves of these lncRNAs. While these lncRNAs possessed little information for segmentation of HSCC and LSCC, they can distinguish the high- and low-risk of death groups perfectly well. In the Kaplan-Meier plot the log-rank p-value is also given. Since the number of HSCC patients included in this study is very small, the log-rank test was based on two groups instead of four groups. LSCC: laryngeal squamous cell cancer; HSCC: hypopharyngeal squamous cell cancer; LR: low-risk group; HR: high-risk group

Among these 9 unique directly-related-to-cancer lncRNAs, only CYTOR and SNHG12 have Wilcoxon test *p*-values < 0.05 (Fig. 5) and may be loosely regarded as differentially expressed genes between LSCC and HSCC subtypes, and between cancer tissues and normal tissues. The small sample size of HSCC in this analysis may explain the results to some degree. Similar to the results of esophageal cancer application, while these 9 lncRNAs cannot distinguish LSCC and HSCC, they do have prognostic value for predicting the risk of death for HNSCC patients (here, LSCC and HSCC were examined together given there were only 6 HSCC patients in this study). Corresponding heat-map and Kaplan-Meier curves are presented in Fig. 6. Lastly, the regulated mRNAs by the identified lncRNAs were retrieved from the lncRNADisease 2.0 database [31] and the pathway enrichment analysis was carried out using the String database [52]. The enriched GO terms and KEGG pathways for these four subtypes are presented in Table 2, from which we observe that no overlaps among these four subtypes occur.

**Table 2:** Enriched pathway analysis for the mRNAs regulated by selected lncRNAs

	GO-BP	GO-CC	GO-MF	KEGG
LSCC	–	–	–	–
HSCC	peptide cross-linking, skin development, epidermis development, keratinization, epithelium development, cornification	cornified envelope		
ESCC	–	nuclear envelope, organelle envelope, organelle, membrane-bounded organelle, intracellular membrane-bounded organelle, organelle part, intracellular organelle part, organelle membrane, intracellular organelle, nuclear part	–	–
EAC	Nephron tubule development	–	–	Nitrogen metabolism

no enriched pathways. *LSCC* laryngeal squamous cell carcinoma, *HSCC* hypopharyngeal squamous cell carcinoma, *ESCC* esophageal squamous cell carcinoma, *EAC* esophageal adenocarcinoma, *GO-BP* gene ontology biological process category, *GO-CC* gene ontology cellular component category, *GO-MF* gene ontology molecular function category, *KEGG* Kyoto encyclopedia of genes and genomes pathways

## Conclusions

The Cox-filter method is among the first efforts to develop feature selection algorithms capable of identifying prognostic genes specifically for different subtypes. When applied to gene expression profiles, it achieved satisfactory performance. In this study, we show that this method is applicable to lncRNA expression profiles, as illustrated by the two real-world applications in which the Cox-filter method identified many lncRNAs with meaningful implication with cancer. The ratio of the two distinct subtypes in these applications represent extreme cases: one with good balance case and one with bad balance. The Cox-filter method can easily deal with the first case. In the second case, it can still estimate the significance level of lncRNAs in minor subtypes by borrowing some information from the dominant subtype. Therefore, the Cox-filter method is a handy tool to construct subtype-specific prognostic lncRNA signatures, indeed.

The big drawback of the Cox-filter method is inclusion of many false positives in the final models. To address this drawback, several extensions that incorporate biological information and prioritize genes with high connectivity levels have been proposed [53, 54]. When applying to lncRNA profiles, the issue is still apparent and thus needs to be addressed as well. However, those extensions cannot be adopted to the lncRNA expression profiles directly because the biological pathway information was retrieved from a knowledgebase such as String [52] or HPRD [55], which focus on mRNAs (protein coding genes). A statistical model (e.g., the WGCNA method [56] with the capacity of constructing co-expression networks/modules is needed before implementing such Cox-filter extensions. Nevertheless, by combining biological relevancy information from the GeneCards database, we further refined the lncRNA lists identified by the Cox-filter method, and the resulting lncRNA signatures have been demonstrated to possess perfect prognostic value.

## Data Availability

Data for head and neck squamous cell carcinoma (the HNSC cohort) were downloaded from The Atlas of ncRNA in Cancer (TANRIC) database (https://www.tanric.org/), and data for esophageal cancer (the ESCA cohort) were downloaded from the Genomic Data Commons of The Cancer Genome Atlas (https://gdc.cancer.gov).

## Abbreviations

ceRNA: Competitive endogenous RNA
EAC: Esophageal adenocarcinoma
ESCC: Esophageal squamous cell carcinoma
FPKM: Fragments per kilo-bases per million
HCC: Hepatocellular carcinoma
HNSCC: Head and neck squamous cell carcinoma
HSCC: Hypopharyngeal squamous cell carcinoma
lncRNA: Long non-coding RNAs
LSCC: Laryngeal squamous cell
TANRIC: The atlas of ncRNA in cancer
TCGA: The cancer genome atlas
